# Low dose of neonicotinoid insecticide reduces foraging motivation of bumblebees

**DOI:** 10.1098/rspb.2018.0506

**Published:** 2018-07-25

**Authors:** Juho Lämsä, Erno Kuusela, Juha Tuomi, Sini Juntunen, Phillip C. Watts

**Affiliations:** 1 Ecology and Genetics, University of Oulu, 90014 Oulu, Finland; 2 Department of Biology, Section of Ecology, University of Turku, 20014 Turku, Finland

**Keywords:** bumblebee, foraging, learning, behaviour, neonicotinoid, imidacloprid

## Abstract

Widespread use of neonicotinoid insecticides, such as imidacloprid, is often associated with diminishing populations of bees; this loss of pollinators presents a concern for food security and may cause unpredictable changes in ecological networks. However, little is known about the potential behavioural mechanisms behind the neonicotinoid-associated pollinator decline. We quantified the effects of low-dose (1 ppb) imidacloprid exposure on the foraging behaviour of bumblebees (*Bombus terrestris*). Individual bumblebees were released into a flight arena containing three patches of robotic flowers whose colour (yellow, orange, blue) indicated whether the flower delivered a reward (sugar solution). Exposure to imidacloprid had no significant effect on measures of bumblebee physical performance (such as flight speed) or learning (identifying rewarding flowers). However, pesticide-treated bumblebees had reduced foraging motivation compared with the control bumblebees, as they visited fewer robotic flowers, were slower to start foraging and did not visit all three flower colours as often. Neonicotinoid concentrations of 1 ppb, often reported in plant nectar near agricultural lands, can thus affect the foraging behaviour of bumblebees. Even without a notable impact on flight performance and learning, a reduction in foraging motivation could explain the poor performance of colonies of bumblebees exposed to neonicotinoids.

## 1. Background

Populations of insect pollinators are declining in many agricultural regions [1,2]. Crop pollination services in Europe and North America are dominated by the honeybee *Apis mellifera* [3–5] and common species of wild bee [6–9], notably bumblebees (*Bombus* spp.) [10,11]. Declining bee populations have generated much concern about maintenance of essential ecosystem services [12–14] and biodiversity [8]. Causes of insect pollinator declines are multifaceted, and include land-use policy, climate change, invasive species and/or the spread of pathogens [1,2,11,14]. One of the most prominent drivers of insect pollinator declines is the use of pesticides for crop protection [2,5,14–16].

Since their introduction in the 1990s, neonicotinoids have become the most widely used class of insecticides globally [5]. Neonicotinoids are absorbed by plants and translocated to tissues, where the pesticide protects against pest damage; neonicotinoids also move to pollen and nectar, thereby allowing oral entry to ‘non-target’ insect pollinators [5,15,16]. In April 2018, the European Union (EU) imposed a total ban on outdoor use of three neonicotinoids (clothianidin, imidacloprid and thiamethoxam), based on a review by the European Food Safety Authority (EFSA) published in February 2018. Most of the available information for the review was on honeybees, with few studies on wild bees. However, the review states that imidacloprid residues in nectar and pollen from treated crops represent a high risk for bumblebees [17]. In June 2018, the EU commission launched a first-ever EU initiative on pollinators. The objectives of the initiative include: (i) improving knowledge of pollinator decline, its causes and consequences; (ii) tackling the causes of pollinator decline; and (iii) raising awareness, engaging society at large and promoting collaboration (http://ec.europa.eu/environment/nature/conservation/species/pollinators/index_en.htm).

One of the key issues regarding the causes of pollinator decline is the sub-lethal effects, such as the foraging behaviour of individual bees, associated with exposure to low doses of neonicotinoids. Concentrations of neonicotinoid pesticide residues in pollen and nectar in treated crops vary considerably, but average maximum values are typically around 2 ppb for nectar and 6 ppb for pollen [15]. However, the experimental studies of pesticide effects on bees typically use higher concentrations of pesticide than typical in the wild [18,19]. Nevertheless, exposure to neonicotinoids is associated with reduced foraging activity in individual bumblebees. For example, worker *Bombus terrestris* exposed to 0.7 ppb imidacloprid dissolved in sugar and 6 ppb imidacloprid on pollen collected less pollen when allowed to forage in the field [20]; also, *B. terrestris* exposed to 2.4 ppb thiamethoxam had longer foraging bouts and returned pollen to the colony less frequently than control bees [21]. Conversely, other studies on *B. terrestris* workers found reduced pollen foraging efficiency at comparatively high (relative to likely residues in crops) doses of neonicotinoid (e.g. 10 ppb imidacloprid [22,23] or thiamethoxam [24]), with exposure to 2.4 ppb of thiamethoxam not affecting individual behaviour (or colony-level function) in *B. terrestris* in one study [24].

In addition to some uncertainty about the dose of pesticide required for a detectable impact, studies on individual bee behaviour rarely quantify variation in foraging actions. Studies on foraging behaviour by individual bees often use data from radio-frequency identification (RFID) tags attached to the thoraxes of bees [20–23]. RFID tags record the movements of individual bees to and from a colony entrance, where reader devices are typically placed. RFID-tag data thus summarize the overall success of foraging bouts (e.g. total time spent away from the hive, amount of pollen collected) [20–23], but not the diversity of actions made away from the reader devices. The importance of quantifying individual actions can be highlighted by two studies [24,25] that used manual observations of free-flying, individual *B. terrestris* to show how exposure to 10 ppb thiamethoxam can alter, for example, flower preference [25] and pollen-collection behaviour [24,25].

As neonicotinoids are potent insecticides, exposure to them has the potential to alter a bee's memory [26–28], foraging efficiency [20–24], flight abilities [29] and initial flower preferences [25], but there has been no systematic examination about which bee behavioural traits might be affected most by exposure to a field-realistic [15,18,19] dose of pesticide. Possible traits affected could include (i) physical performance (e.g. flight speed, distance covered during foraging), (ii) learning (e.g. avoidance of non-rewarding food sources) and (iii) motivation (time taken to start foraging, curiosity to explore novel food sources). Studies on the behaviour of individual bees are required to identify the underlying mechanisms behind the widely reported declines in the general performance of bee colonies exposed to neonicotinoid [5,15,16]. In particular, foraging behaviour is perhaps the defining aspect of bee life history, with effective foraging behaviour essential to deliver sufficient resources for colony growth and queen production.

We hypothesize that exposure to a low dose of neonicotinoid insecticide will reduce bumblebee foraging capability. To test this hypothesis, we quantified the effects of low-dose (1 ppb in nectar and none in pollen) exposure to neonicotinoid (imidacloprid) on the foraging behaviour of the bumblebee *B. terrestris*. Individual bumblebees were released to a flight arena equipped with robotic flowers that differed in their colour and food availability (sugar/quinine), thereby allowing an examination of both learning and foraging ability.

## 2. Methods

### (a) Training of bumblebees

**Figure 1.**
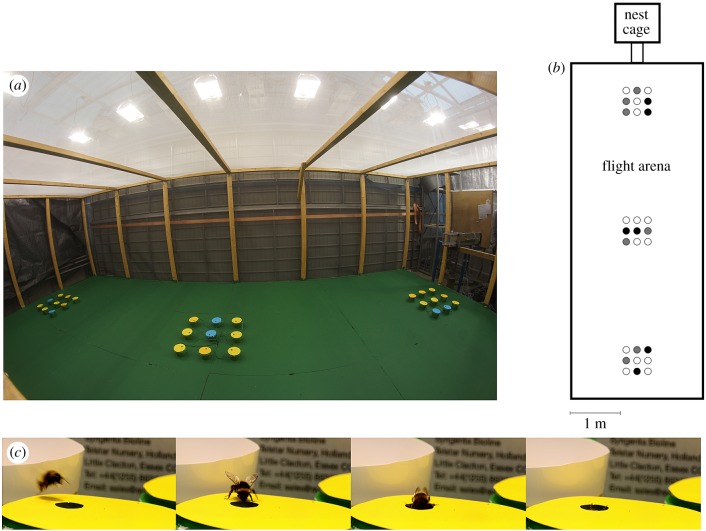
(*a*) Photograph and (*b*) schematic diagram of the experimental design of the flight arena used to quantify bumblebee foraging behaviour. Size of robotic flowers in (*b*) is only illustrative. White circles represent yellow (rewarding) flowers, while grey and black circles represent orange and blue (both punishing) flowers. (*c*) Time series photographs of foraging by a bumblebee on a robotic flower (during training in the nest cage). Photo (*a*) credit: Lassi Kalleinen; photo (*c*) credit: Kari Saikkonen.

Naive bumblebees (*Bombus terrestris*, L) were imported from Syngenta Bioline, UK, by Puutarhaliike Helle OY (Lieto, Finland). First, the sugar tank was removed (provided by the producer) to motivate the bumblebees to forage and to have total control of their diet. Bumblebee colonies were placed in a nest cage (0.62 × 0.75 × 0.8 m) equipped with five robotic flowers (figure 1; electronic supplementary material, figure S2) [30], three gravity feeders and a cup that provided pollen (collected from honeybees, imported by CoCoVi, Kihniö, Finland). Gravity feeders and robotic flowers provided 35% (w/w) sucrose solution. To familiarize the bumblebees to differently coloured flowers, all robotic flowers differed in colour (three yellowish and two bluish hues; see electronic supplementary material for details). The naive bumblebees thus learnt to feed from the artificial flowers. The nest cage was connected to the (6.2 × 2.4 × 1.6 m) flight arena via a closable Plexiglas tube (figure 1).

### (b) Experimental design

Our experiment was designed to identify whether any aspects of bumblebee foraging behaviour are affected by exposure to a field-realistic, low-dose (1 ppb) exposure to imidacloprid. For individual bumblebees, we measured (i) physical performance (speed of movements between flowers, linear distance moved between flowers), (ii) the ability to differentiate between rewarding and non-rewarding flowers based on the colour of the flowers, and (iii) foraging motivation (time taken until first flower visitation, number of flowers visited, willingness to try foraging from flowers of all colours). To achieve this, bumblebee colonies were assigned into two treatments: (i) those exposed to imidacloprid and (ii) control colonies not exposed to the pesticide. Control colonies were provided with sucrose solution (35% w/w), while bumblebees in the pesticide treatment were fed with similar 35% (w/w) sucrose solution containing 1 ppb imidacloprid (commercial product Confidor). Pollen was not treated with imidacloprid. Note that bumblebees apparently cannot taste imidacloprid, but favour the neonicotinoid-treated food if given an equal choice [31]. Imidacloprid was not used in the flight arena. We did not measure the accumulated dose of imidacloprid for each bumblebee. However, bumblebees fed 2.1 ppb imidacloprid in sugar solution accumulate 4–10 nM pesticide within their brains during a 3-day period [32].

Experiments began after one week of training (in the nest cage only) to feed from the robotic flowers, with simultaneous imidacloprid exposure for the pesticide treatment. To collect data on foraging behaviour, an individual bumblebee was released into the flight arena and allowed to forage in the novel environment. Foraging was considered finished when the bumblebee had visited at least one robotic flower and subsequently stopped foraging and flying for a minimum of 5 min. The bumblebee was then removed from the flight arena (an individual bee was not used twice). Bumblebees had no prior experience of the flight arena (i.e. the spatial pattern of flowers in three patches) or of the punishing quinine treatment prior to data collection, but the three colours used there were also present in the nest cage.

### (c) Experimental set-up

The experiments were completed during two years, in March–April of 2015 and March–April of 2016. Foraging data were collected using a computer-controlled robotic flower system that automatically registered all foraging events [30] (figure 1; electronic supplementary material, figure S2). The flight arena contained 27 robotic flowers arranged as three patches of nine flowers. The distance between adjacent flowers within a patch was 20 cm, and the distance between patches was 200 cm (figure 1). Fourteen flowers provided 35% (w/w) neonicotinoid-free sucrose solution (reward) and 13 flowers provided 0.12% (w/w) quinine solution without sugar (punishment). Rewarding flowers were yellow, while the non-rewarding (punishment) flowers were orange or blue to provide a source of relatively difficult (orange, *n* = 7 flowers, two or three flowers per patch) or easy (blue, *n* = 6 flowers, two flowers per patch) decisions (see below for colour modelling) to discriminate between rewarding and non-rewarding flowers. The size of the feeding cup in each flower was 1.7 µl. In the flight arena, every cup was filled regularly every 5 min for all flowers simultaneously.

The hexagonal colour distance [33] between yellow and orange flowers was 0.076 hexagonal units, which is just above the level (approx. 0.05) that the bumblebees can discriminate visually [34]; blue flowers were approximately 0.4 hexagonal units from the yellow and orange flowers, and thus could be readily discriminated from either yellow or orange flowers by bumblebees (figure 1*a*; electronic supplementary material, figure S1 and table S1). Colour distances were measured on the basis of spectrometer measurements of flower colours (Ocean Optics USB 3000) and modelling using custom R code (Klaus Lunau 2014, personal communication) that included the sensitivity functions of *B. terrestris* photoreceptors. Robotic flowers differed only in their colour and in the delivery of a reward or punishment solution.

### (d) Statistical analyses

The robotic flower software [30] recorded each visit and its duration to any of the artificial flowers because an individual bumblebee was released to the flight arena. We could thus analyse (i) the entire foraging sequence (consisting of multiple records per individual) and (ii) a summary of foraging events, constrained to one value per individual. Depending on the probability distribution of each independent variable, either linear mixed models (LMMs) or generalized linear mixed models (GLMMs) were fitted. Data with multiple values per individual represented learning behaviours, while data with one value per bumblebee included analyses of physical performance and foraging motivation (table 1). In the learning analyses with multiple values per individual, the random factor was hierarchical (individual bumblebee nested within the colony). For other analyses, bumblebee colony was included as a random factor. All independent variables were analysed as separate models, with treatment as a fixed factor, except in the learning analyses, where we fitted learning rate as a fixed factor.

**Table 1.** Summary of LMM and GLMM analyses on the effect of pesticide (1 ppb imidacloprid) treatment on bumblebee foraging behaviour. Each result is from a separate model and the structure of the models was always similar: independent variable ∼ fixed factor + random factor. The estimate, standard error (s.e.) and *p*-values are for the fixed factor, which is treatment (control/imidacloprid) in categories 1 (physical performance) and 3 (foraging motivation). In category 2 (learning), the fixed factor is the interaction of treatment and visit sequence (1st visit to any flower, 2nd visit, etc.), representing the learning curve. Bumblebee colony is the random factor in categories 1 and 3, while the individual bumblebee nested within the colony is used as the random factor for category 2.

independent variable	estimate	s.e.	*p*-value
1. physical performance			
average speed of movements between flowers (cm s^−1^)	−0.385	0.657	0.586
total distance moved between flowers (cm)	−419.9	323.7	0.259
average time [log(*s*)] spent feeding	−0.013	0.061	0.831
2. learning			
rewarding (yellow) versus punishing (blue and orange) flowers	−0.004	0.005	0.407
easy discrimination task: yellow versus blue flowers	0.015	0.014	0.256
difficult discrimination task: yellow versus orange flowers	−0.007	0.005	0.149
3. foraging motivation			
time [log(*s*)] taken until first flower visitation	0.692	0.123	<0.001
duration (s) of foraging period	−473.2	187.5	0.061
number of flowers visited	−0.166	0.080	0.037
all flower types visited (0 = no, 1 = yes)	−1.462	0.352	<0.001
any blue (punishing) flowers visited (0 = no, 1 = yes)	−1.371	0.396	<0.001

In analyses of avoidance learning behaviour (i.e. the rate at which bees would learn to avoid the non-rewarding orange and blue flowers), we included an interaction term of visit sequence (1st visit to any flower, 2nd visit, etc.) and treatment as a fixed factor, to examine whether the slope of the learning curve was different between control and pesticide treatments. For this examination of ‘avoidance learning’, we discriminated between (i) an easy task (visiting yellow rather than blue flowers) and (ii) a comparatively more difficult task (discriminating between yellow and orange flowers).

All learning analyses, and analyses whether all colours were visited, and subsequently whether any blue flowers were visited, had a binary response variable, so a logistic GLMM was fitted. Number of flowers visited is a count variable, which featured over-dispersion, so a GLMM with negative binomial distribution was used in the analyses. Variables in the physical performance section, and analyses on time until first flower visitation and duration of foraging period, were continuous variables for which LMMs were used. We checked the residual distribution for each model and used QQ-plots for the LMMs to investigate model diagnostics. Because of uneven residuals and skewed QQ-plots, a log transformation was used for average time (s) of feeding, and time (s) taken until first flower visitations in table 1. Basic model diagnostics for table 1 are in the electronic supplementary material.

**Figure 2.**
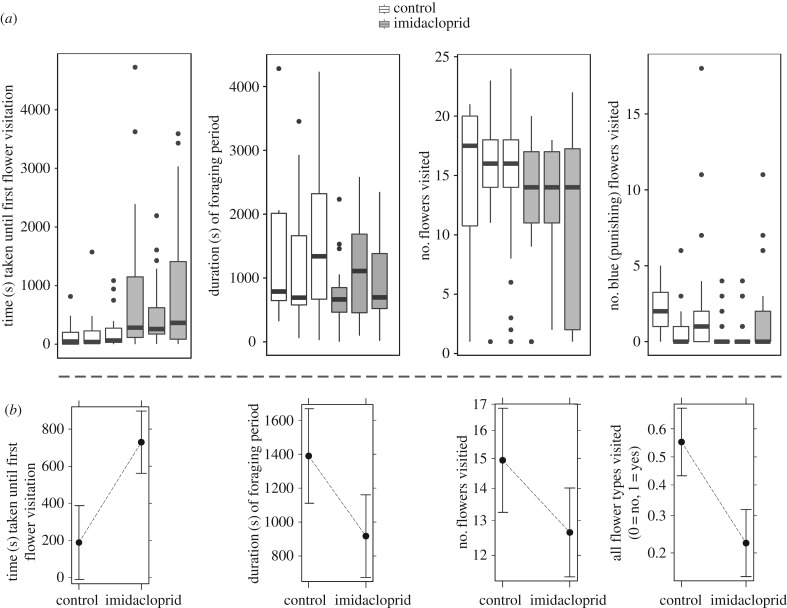
Effect of 1 ppb imidacloprid treatment on bumblebee foraging behaviour. (*a*) Description of data (one value per bumblebee) with bumblebee colonies on the *x*-axis. (*b*) Treatment (control/imidacloprid) effect plots from LMM and GLMM modelling results related to the boxplots above. In (*a*), the black line indicates the median, boxes outline the 25th and 75th percentiles, and whiskers represent 1.5 times the interquartile range with data points outside that range marked as dots. In (*b*), black dot represents the treatment mean and the whiskers represent an estimation of the 95% confidence intervals for the mean value.

**Figure 3.**
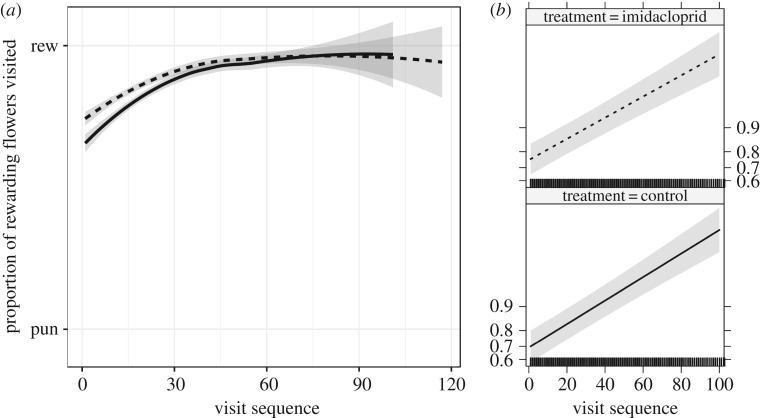
Effect of imidacloprid treatment on bumblebee's discrimination learning (*n* = 65 control bees and 94 treatment bees, 6644 total visits). (*a*) The average proportion of all rewarding and punishing flowers visited by visit sequence (1st visit, 2nd visit, etc.) and s.e. for the mean (shaded area). (*b*) Effect plots from logistic GLMM analyses (see Methods and Results for details). Dashed line indicates 1 ppb imidacloprid treatment and solid line is the control.

Statistical analyses were completed in R v. 3.4.3 [35] using LME4 v. 1.1-15 [36] with statistical significance of models derived from LMERtest v. 2.0-36 [37]. Residual diagnostics and overdispersion analyses of GLMMs were completed with DHARMa v. 0.1.6 [38]. Treatment effect plots in figures 2 and 3 were plotted with the package effects v. 4.0-0 [39]. Other plots were arranged with ggplot2 v. 2.2.1 [40].

## 3. Results

We measured the foraging behaviour of 159 bumblebees (65 control bees and 94 pesticide treatment bees), from six colonies (three control and three pesticide treatment colonies), to identify whether exposure to a low (1 ppb) dose of imidacloprid affected different aspects of foraging behaviour. Over a total foraging period of 70 h, these bumblebees made 6644 visits to the robotic flowers, spent about 6 h in total feeding and moved a total linear distance of 3.9 km from flower to flower while foraging.

We found no obvious decline on bumblebee physiological performance in the flight arena associated with the low-dose imidacloprid exposure. Hence, pesticide treatment had no statistically significant effect on the average speed of movement between flowers (on average 4.7 cm/s in control and 4.4 cm s^−1^ in the pesticide group, estimate: −0.385, s.e.: ±0.657), total distance moved between flowers (on average 2800 cm in the control and 2400 cm in the pesticide group, estimate: −419.9, s.e.: ±323.7) and the average time (s) spent feeding (on average 3.2 s in both the control and the pesticide group, estimate: −0.013, s.e.: ±0.061, table 1).

By contrast, many behaviours associated with foraging motivation were significantly affected by the pesticide treatment (table 1). Bumblebees exposed to imidacloprid were significantly slower (on average 188 s in the control and 728 s in the pesticide group) to make their first visit to an artificial flower (LMM, estimate for the log-transformed model: 0.692, s.e.: ±0.123, *p* = 8.32 × 10^−8^). Despite the later start in foraging, the time of the last recorded visit to a flower was not significantly later (on average 1540 s in the control and 1619 s in the pesticide group; LMM, estimate: 79.78, s.e.: ±166.70, *p* = 0.633). Accordingly, there is some indication that bumblebees exposed to imidacloprid had shorter foraging periods (1394 s versus 911 s), with foraging duration (the time of last visit to a flower subtracted by the time of first visit) approaching statistical significance (LMM, estimate: −473.2, s.e.: ±187.5, *p* = 0.061). Further evidence of a reduction in foraging motivation in the pesticide-treated bumblebees was apparent by visiting fewer flowers (on average 12.6 visitations versus 14.9 visitations; GLMM, estimate via the log link function: -0.166, s.e.: ±0.080, *p* = 0.037; table 1 and figure 2.).

Pesticide treatment also had a significant effect on the pattern of foraging, with bumblebees in the pesticide treatment failing to visit all three flower colours as often as bumblebees from the control group (GLMM, estimate via the logit link function: −1.462, s.e.: ±0.352, *p* = 3.19 × 10^−5^): more than half (55%) of the bumblebees from the control group visited all three flower colours, compared with less than a quarter (22%) of bumblebees from the imidacloprid treatment. An apparent disinterest in visiting the blue (non-rewarding) flowers drove this effect, as the bumblebees exposed to imidacloprid failed to visit the blue flowers more often than bumblebees from the control treatment (GLMM, estimate via the logit link function: −1.371, s.e.: ±0.396, *p* = 5.32 × 10^−4^; figure 2, table 1). Only 27% of the bumblebees in the pesticide treatment group visited blue flowers at least once, compared with 58% of the bumblebees from the control group. By contrast, most bumblebees from both treatments visited an orange (also non-rewarding) flower at least once (94% and 95% of bumblebees from the pesticide and control treatments respectively).

Imidacloprid treatment had no significant effect on our measures of learning behaviour, as apparent from the models that examined the pattern of visits to rewarding and non-rewarding flowers explained by the interaction of treatment and visit sequence as individual level GLMMs (estimate (via the logit link function): −0.004, s.e.: ±0.005, *p* = 0.407, table 1 and figure 3, treatment without the interaction from the same model: estimate (via the logit link function): 0.296, s.e.: ±0.385, *p* = 0.442). While there is some indication of a difference between treatments during the beginning of the foraging bout of the learning curve (figure 3), excluding the later (i.e. after 30) visits to flowers did not yield a statistically significant effect of pesticide treatment on avoidance learning (i.e. the interaction of treatment and visit number is still statistically not significant (GLMM, estimate via the logit link function: −0.018, s.e.: ±0.010, *p* = 0.079; treatment without the interaction from the same model: estimate via the logit link function: 0.451, s.e.: ±0.370, *p* = 0.223). Further evidence that low-dose exposure to imdacloprid had little impact on learning was derived from the separate analyses of foraging on non-rewarding (orange and blue) flowers that differed in colour. Slopes of the learning curve in the easy task (yellow versus blue, GLMM, estimate via the logit link function: 0.015, s.e.: ±0.014, *p* = 0.256) and in the difficult task (GLMM, estimate via the logit link function: −0.007, s.e.: ±0.005, *p* = 0.149) were both not statistically significant.

## 4. Discussion

A substantial amount of evidence [4,5,15,16] shows the negative effects of neonicotinoids on non-target organisms, such as wild bee pollinators. However, there is a gap in our knowledge on the actual behavioural mechanisms causing the reduction in foraging success of bee pollinators. Here, we show that a low, field-realistic dose (1 ppb imidacloprid in sugar solution, and no pesticide in pollen) of imidacloprid affects the foraging behaviour of individual bumblebees. Neonicotinoid exposure at this low concentration had no detectable effect on measures of physical performance and ability to learn to discriminate among flower colours, but rather reduced the motivation of individual bumblebees to forage *per se*. These data imply that concentrations of pesticide at which bumblebees might be apparently healthy (e.g. from flight performance) are not equivalent to the concentrations at which there is a lack of impact on foraging success. A subtle reduction in the individual bumblebee's motivation for foraging can very well scale up to a decline at the population level, even without a notable increase in lethality, or decrease in physical performance caused by the pesticides.

### (a) Foraging behaviour: motivation and physiology

Exposure to neonicotinoids alters foraging efficiency in bumblebees. For example, workers spend more time on foraging journeys [21,22] and collect less pollen [20–24]. A key implication of our data is that the increased time spent foraging in these experiments is probably not derived from impaired flight performance. Rather, exposure to imidacloprid reduces bumblebee foraging motivation. The qualitative reduction in foraging period in the pesticide treatment implies that bumblebees do not compensate for the slow start to a foraging bout by subsequently foraging for longer. As the bumblebees in the imidacloprid treatment are visiting fewer flowers and spending their time on something other than active foraging, they are spending energy for their own metabolism instead of bringing food for their colony. The cumulative effects of these small individual actions probably scale to poor colony-level efficiencies observed in other studies. Our data thus shed light on the reasons behind reduced foraging efficiency [20–23] measured with an RFID system, or poor colony performance [41,42] of bumblebees exposed to neonicotinoids. Our study is consistent with other work that found a reduction in flight duration (−54%), distance (−56%) and average velocity (−7%) of honeybees exposed to sub-lethal doses (1.96–2.90 ng per bee per day) of thiamethoxam [29]. However, our methods were different, as our bumblebees were allowed to actively forage, while the honeybees [29] were immobilized into an experimental ‘flight mill’ (i.e. they were not foraging during measurements).

### (b) Foraging: learning

Given the importance of effective foraging for the health of bee colonies, it is unsurprising that bumblebees are capable of learning to solve complex tasks [43,44]. Neonicotinoids bind to the acetylcholine receptors of the nervous system [26], providing a route for impaired learning ability in bumblebees exposed to neonicotinoids [28]. That our low-dose imidacloprid treatment had no significant effect on the ability of bumblebees to differentiate even between subtle differences in flower colour (i.e. between rewarding yellow and non-rewarding orange flowers) associated with resource acquisition presents a contrast with experiments based on the classical proboscis extension reflex (PER) in both bumblebees [28] and in honeybees [27] exposed to neonicotinoids. One explanation may be in the very different methodology used, in addition to higher doses of neonicotinoids used in previous work (2.4 ppb of thiamethoxam [28] and 10 and 100 ppb of imidacloprid [27], respectively). The PER studies use immobilized animals whose antennae are stimulated with sugar solution, causing their tongues to stick out as a reflex, whereas our bumblebees were free-flying and could choose the flowers they visited.

The relationship between discrimination learning and foraging success is complex. For example, one study reported an intuitive positive correlation between learning rate and nectar foraging rate in bumblebees [45]; by contrast, other studies on bumblebees did not find such a correlation, but rather showed that more ‘error-prone’ bumblebees acquired more resources during their lifetimes [46] and discovered novel food sources faster than more accurate individuals [47]. Irrespective of the potential association between learning and foraging success, our study did not find a neonicotinoid-associated reduction in learning abilities; the implication is that a reduction in discrimination learning ability is not likely to be a principal cause of the observed decline in colony performance in bumblebees exposed to similar concentrations of imidacloprid [5,15,16].

### (c) Foraging curiosity

As the bumblebees in the pesticide treatment tended not to visit any blue flowers (i.e. visits to at least one blue flower, table 1, figure 2), the avoidance is unlikely to represent a component of learning behaviour. Yellowish flowers (i.e. orange and yellow) were dominant (21 out 27 flowers; figure 1) in the flight arena, with the rarer blue flowers presenting an obvious contrast. Hence, avoidance of blue flowers may be interpreted as reduced foraging motivation or a reduction in ‘curiosity’ to explore a rarer flower type. An implication of this result is that exposure to imidacloprid might lead to a reduced curiosity to seek novel food sources.

Of course, many plant assemblages (i.e. flower types) vary spatially and temporally. As such, behavioural flexibility might allow efficient exploitation of diverse resources, and a bias caused by neonicotinoids towards avoiding exploration of potential food sources could limit resources acquisition, with possible population-level effects for bees. Our data somewhat contrasts with a study [24] where bees fed with 10 ppb of thiamethoxam increased the rate of switching between different varieties of apple blossoms, although in that study, all apple blossoms were rewarding and supposedly quite similar in colour, whereas our artificial flowers were rewarding/punishing and varied considerably in colour (yellow versus blue). Neonicotinoids also alter bumblebee preferences for real flowers [25], which could cause unpredictable changes in ecological networks, in combination with the reduced curiosity observed in this study.

### (d) Field realism

Many experiments that quantify the potential impacts of neonicotinoids on bee colony viability and individual bee behaviour have been criticized for exposing bees to unrealistically high doses [5,15,19]. Concentrations of neonicotinoid pesticide residue in pollen and nectar in seed treated crops vary considerably, but average *maximum* values (from 20 published studies) are around 2 ppb for nectar and 6 ppb for pollen [15]. We found significant effects on bee foraging at 1 ppb of imidacloprid in nectar and none in pollen, a concentration which can be stated as firmly field realistic, rather than a ‘worst case scenario’ [19].

A notable exception for criticism on concentrations used [15,19] is a study [41] that detected reduced colony growth and queen production in *B. terrestris* fed with low doses (0.7 and 1.4 ppb) of neonicotinoid in sugar solution, albeit with the bumblebees also fed with neonicotinoid-treated (6 and 12 ppb) pollen. Addition of pesticide in pollen makes a comparison with our study less straightforward, as we did not treat pollen with pesticide. In addition, another study [48] found no effect of 2 ppb imidacloprid on feeding activity in *B. terrestris*, while exposure to 10 and 20 ppb pesticide failed to gather any behavioural data due to lethality or total passivity of the worker bumblebees. The magnitude of the effects associated with exposure to pesticides thus depend on the concentration and the duration of pesticide treatment, and whether bees were given a choice about whether to take pesticide-treated food or they could also take uncontaminated food [19]. The considerable variation in experimental design among studies makes direct comparisons difficult.

Laboratory experiments inevitably lack field realism, but are the only practical way to partition components of bee foraging behaviour. Obtaining detailed behavioural data from free-flying, foraging bees is technically difficult even in the laboratory, and few studies have quantified the effects of pesticides on foraging patterns of individual bees. Our results highlight the need for studies with accurate measurements on low chronic/semi-chronic pesticide concentrations, to identify the threshold(s) where the effects of pesticides have a significant impact on foraging by different bee species. Similar concentrations of imidacloprid used in this study are commonly measured from plant nectar and pollen [5,15], and from pollen collected by honeybees [49,50], so the reduced foraging motivation observed in this study may already be occurring for the wild bees in the agriculturally intensive regions of the world.

## 5. Conclusion

Our experiment is one of the first to combine direct measurements of several behavioural traits of free-flying bumblebees exposed to a field-realistic, sub-lethal dose of neonicotinoid insecticide. Exposure to imidacloprid did not elicit a reduction in physical performance indicators (such as flight speed, linear distance covered) or learning, but did reduce the foraging motivation (and potentially the curiosity to try different food sources); this reduction in motivation may account for the negative colony effects observed in many studies. Therefore, when evaluating the effects of neonicotinoids on non-target species, a lack of detectable effects on physical performance cannot be viewed as evidence that the current practices have little or no impact on bee foraging.

## Supplementary Material

Supplementary material from “Low dose of neonicotinoid insecticide reduces foraging motivation of bumblebees”

## Data accessibility

Data are deposited in the Dryad Digital Repository at http://dx.doi.org/10.5061/dryad.vb06d3k [51].
