# Supplementary material for: Low dose of neonicotinoid insecticide reduces foraging motivation of bumblebees
Source: Proc Biol Sci. 2018 Jul 25;285(1883):20180506. doi: 10.1098/rspb.2018.0506 (PMC6083263; doi:10.1098/rspb.2018.0506)
Supplement: Supplementary material from “Low dose of neonicotinoid insecticide reduces foraging motivation of bumblebees” [file rspb20180506supp1.pdf]

Supplementary material from “Low dose of neonicotinoid insecticide reduces foraging motivation of bumblebees”

Proceedings of the Royal Society B
doi: 10.1098/rspb.2018.0506

Juho Lämsä[1,*], Erno Kuusela[1], Juha Tuomi[1,2], Sini Juntunen[1] & Phillip C. Watts[1]

[1]Ecology and Genetics, University of Oulu, 90014 Oulu, Finland

[2]Department of Biology, Section of Ecology, University of Turku, 20014 Turku, Finland

*email: juho.lamsa@oulu.fi

Supplementary Methods

Paint details

Covers and the other outside parts of the flower robots were painted green (Maston’s Spraypaint 100 Green, hue 802, RAL 6029) to closely match the green colour of the flying arena floor (Teknos’s Interior paint, matte, BIORA 20, hue IN2-RAL-6029). All cables of robotic flowers were painted green with the same product as the flying arena floor (Teknos’s Interior paint, matte, BIORA 20, hue IN2-RAL-6029). Robotic flowers were refilled and cleaned at four day intervals, and the colour discs were replaced at the same time.

Colour modelling details

Test colours/hues were printed by an inkjet printer (Stylus Photo PX720WD, Epsom) on a film paper (Canon, matte) to minimise effects of light reflection. Degree of hue similarity was measured using

a reflectance spectrophotometer (USB 3000, Oceans objectives), which was attached to a supportive stem to minimise the disturbance that a shadow would have caused for the results. Spectral data were recorded from a specific area (*ca*. 5 cm diameter) that was defined using a black plastic tube (43 mm long, attached to spectrophotometer) that reduced potential noise and increased the number of photons. All readings were taken in quantums. For the background spectrum, the green floor of the flying arena was used to create the correct contrast.

Finally, the chosen hexagonal distance between yellow and orange was 0.07 hexagonal units, between yellow and blue 0.46 hexagonal units and between orange and blue 0.42 hexagonal units (Chittka, 1992; Dyer & Chittka, 2004) (see Fig S1 & Table S1). After modelling bumblebee trichromatic colour vision, suitable hues (MS Paint colour coding was used) for the robotic flowers were: (1) HUE 43, luminance 120 for bright yellow, (2) HUE 35, luminance 120 for orange, (3) HUE 135, luminance 140 for blue, (4) HUE 30, luminance 120 for dark orange and (5) HUE 145, luminance 140 for dark blue. The saturation for all hues was 240. Colours 1, 2 and 3 were used in the flight cage; others were present only in the training process in the nest cage.

Details of experiment room

The flight arena was constructed with a wooden frame and walls from a plastic insect net. Temperature was 20°C. Natural light was blocked with tarps both inside and outside the greenhouse and illumination was provided by fourteen high-pressure metal halide lamps (400 W, Phillips HPI-T Plus) that were lit between 8 am to 8 pm.

Pesticide treatment details

Due to the low concentration of imidacloprid used in the experiment, a base solution of 1 liter containing 0.242 g Confidor (70 % imidacloprid) was made. 1 ml of base solution was inserted to a

canister containing 17 kg of 35 w/w % sucrose solution to achieve the concentration of 1 ppb. Before refilling of the artificial flowers with fresh sucrose solution, the canister was shaken thoroughly.

Animal welfare

The bumblebees were treated as well as possible during the experiments. Food (sugar solution and pollen) was provided regularly and unnecessary suffering was avoided. After data collection each bumblebee was taken to freezer, which was considered the most humane way available of putting down the animals. The same procedure was performed to the bumblebee colonies. All actions were performed under Finnish law of test animals (Act on the Use of Animals for Experimental Purposes, 62/2006):

http://www.finlex.fi/fi/laki/kaannokset/2006/en20060062.pdf

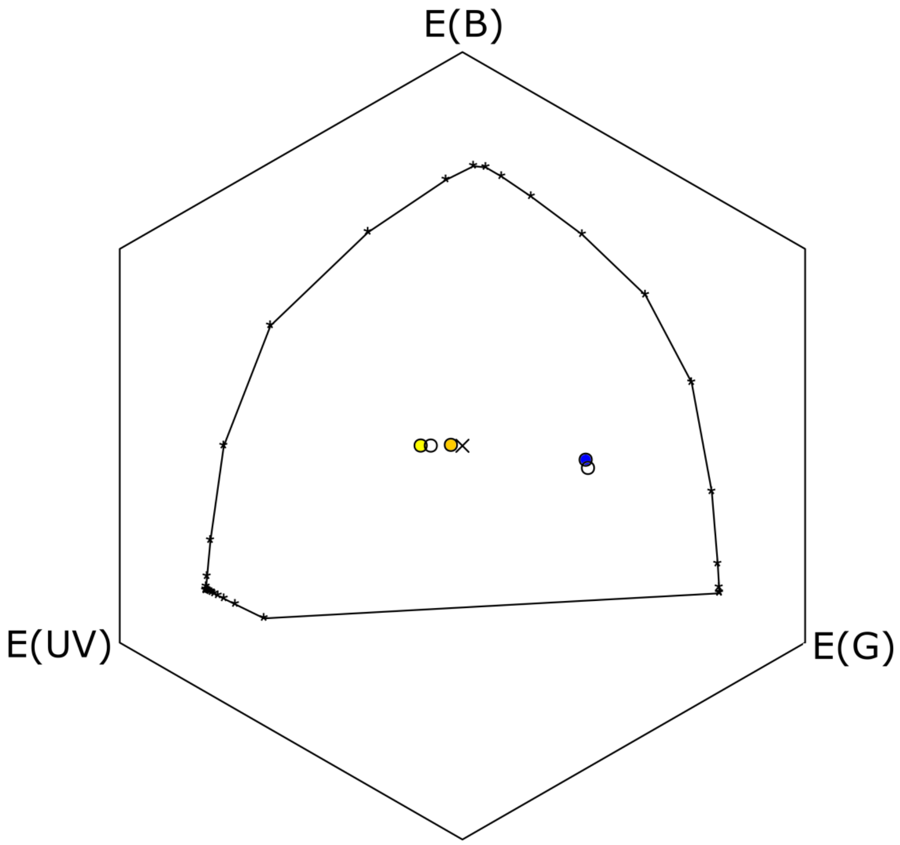

Fig. S1. Colour hexagon of the symbol colours used. The coloured ones were used in the actual experiment in flight arena and the empty circles were present only in training phase inside the nest cage.

Table S1. Results of colour modelling. Hue43yellow is the rewarding colour in the flight arena. Hue35yellow and hue130blue are the punishing flowers in the flight arena. Others were present only in the nest cage.

```
Excitation values:
Stimulus hue30yellow : E(UV)=0.474122   E(B)=0.428454   E(G)=0.381669
Stimulus hue35yellow : E(UV)=0.447475   E(B)=0.387157   E(G)=0.325573
Stimulus hue43yellow : E(UV)=0.421573   E(B)=0.407127   E(G)=0.387857
Stimulus hue130blue  : E(UV)=0.539714   E(B)=0.684198   E(G)=0.899302
Stimulus hue135blue  : E(UV)=0.507667   E(B)=0.634614   E(G)=0.873964

Distances:
      [,1]                       [,2]                       [,3]
 [1,] "hue30yellow"        "hue35yellow"            "0.025505"
 [2,] "hue30yellow"        "hue43yellow"            "0.050901"
 [3,] "hue30yellow"        "hue130blue"             "0.393118"
 [4,] "hue30yellow"        "hue135blue"             "0.401323"
 [5,] "hue30yellow"        "origin"                 "0.080068"
 [6,] "hue35yellow"        "hue30yellow"            "0.025505"
 [7,] "hue35yellow"        "hue43yellow"            "0.076393"
 [8,] "hue35yellow"        "hue130blue"             "0.41853"
 [9,] "hue35yellow"        "hue135blue"             "0.426596"
[10,] "hue35yellow"        "origin"                 "0.105573"
[11,] "hue43yellow"        "hue30yellow"            "0.050901"
[12,] "hue43yellow"        "hue35yellow"            "0.076393"
[13,] "hue43yellow"        "hue130blue"             "0.342694"
[14,] "hue43yellow"        "hue135blue"             "0.351345"
[15,] "hue43yellow"        "origin"                 "0.029298"
[16,] "hue130blue"         "hue30yellow"            "0.393118"
[17,] "hue130blue"         "hue35yellow"            "0.41853"
[18,] "hue130blue"         "hue43yellow"            "0.342694"
[19,] "hue130blue"         "hue135blue"             "0.021684"
[20,] "hue130blue"         "origin"                 "0.313408"
[21,] "hue135blue"         "hue30yellow"            "0.401323"
[22,] "hue135blue"         "hue35yellow"            "0.426596"
[23,] "hue135blue"         "hue43yellow"            "0.351345"
[24,] "hue135blue"         "hue130blue"             "0.021684"
[25,] "hue135blue"         "origin"                 "0.322163"
```

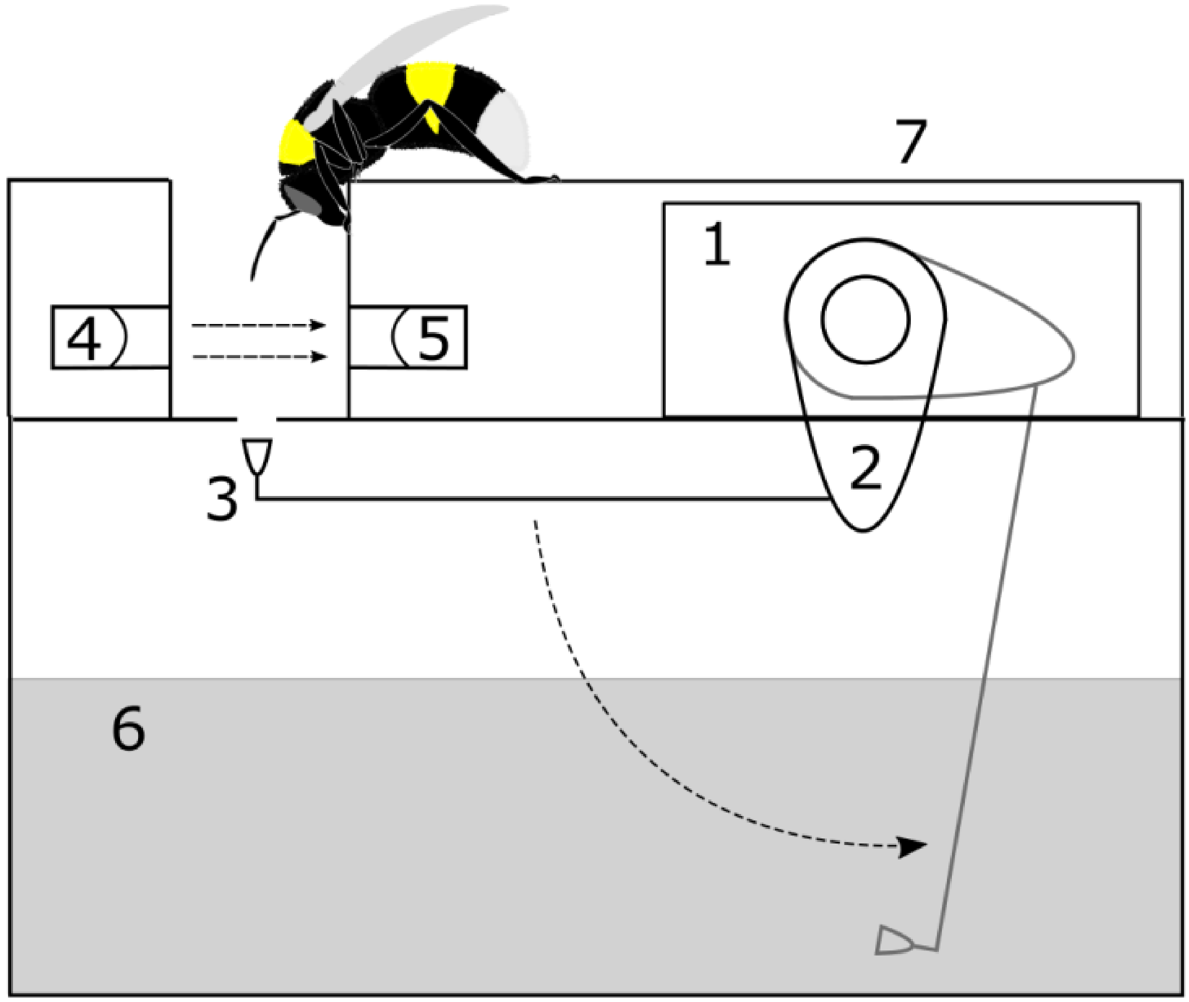

Fig. S2. Schematic view of robotic flower. 1. Servo. 2. Servo arm. 3. Nectar cup (in the feeding position, movement range represented by the curved dashed arrow). 4. IR LED (light path represented by two dashed arrows). 5. IR phototransistor. 6. Nectar container. 7. Top cover. Figure was published in Ecology & Evolution under Creative Commons Attribute License by Kuusela & Lämsä (2016, link to the article: http://onlinelibrary.wiley.com/doi/10.1002/ece3.2062/full)

Supplementary results

Table S2. Additional summary data by colonies.

	date	Count of bee	average speed of movements between flowers (cm/s)		
			Average	StdDev	SE
**control**		**65**	**4.70**	**1.50**	**0.19**
nest 3	March 2015	8	4.80	1.29	0.46
nest 4	March 2015	23	4.66	1.36	0.28
nest 8	March 2016	34	4.70	1.66	0.28
**imidacloprid**		**94**	**4.37**	**2.13**	**0.22**
nest 6	April 2015	37	5.51	1.98	0.33
nest 9	March 2016	33	3.64	1.39	0.24
nest 10	April 2016	24	3.79	2.57	0.52
**Grand Total**		**159**	**4.50**	**1.90**	**0.15**

	date	Count of bee	total distance moved between flowers (cm)		
			Average	StdDev	SE
**control**		**65**	**2834.54**	**1836.08**	**227.74**
nest 3	March 2015	8	2716.33	1967.06	695.46
nest 4	March 2015	23	2830.69	1746.54	364.18
nest 8	March 2016	34	2863.05	1924.99	330.13
**imidacloprid**		**94**	**2418.08**	**1769.19**	**182.48**
nest 6	April 2015	37	2890.88	1887.12	310.24
nest 9	March 2016	33	2191.28	1450.37	252.48
nest 10	April 2016	24	2049.09	1939.19	395.84
**Grand Total**		**159**	**2588.58**	**1802.46**	**142.94**

	date	Count of bee	average time (s) spent feeding		
			Average	StdDev	SE
**control**		**65**	**144.98**	**85.91**	**10.66**
nest 3	March 2015	8	169.25	121.89	43.09
nest 4	March 2015	23	145.48	68.90	14.37
nest 8	March 2016	34	138.94	88.42	15.16
**imidacloprid**		**94**	**124.44**	**102.44**	**10.57**
nest 6	April 2015	37	111.41	62.54	10.28
nest 9	March 2016	33	106.70	95.52	16.63
nest 10	April 2016	24	168.92	144.32	29.46
**Grand Total**		**159**	**132.84**	**96.28**	**7.64**

	date	Count of bee	average time [log(s)] spent feeding Average	StdDev	SE
**control**		**65**	**2.01**	**0.51**	**0.06**
nest 3	March 2015	8	1.94	0.83	0.29
nest 4	March 2015	23	2.08	0.36	0.07
nest 8	March 2016	34	1.98	0.52	0.09
**imidacloprid**		**94**	**1.88**	**0.56**	**0.06**
nest 6	April 2015	37	1.87	0.57	0.09
nest 9	March 2016	33	1.89	0.37	0.06
nest 10	April 2016	24	1.88	0.76	0.15
**Grand Total**		**159**	**1.93**	**0.54**	**0.04**

	date	Count of bee	time (s) taken until first flower visitation Average	StdDev	SE
**control**		**65**	**188.15**	**290.48**	**36.03**
nest 3	March 2015	8	188.13	302.14	106.82
nest 4	March 2015	23	190.09	331.80	69.18
nest 8	March 2016	34	186.85	266.24	45.66
**imidacloprid**		**94**	**728.05**	**952.81**	**98.27**
nest 6	April 2015	37	787.05	1076.36	176.95
nest 9	March 2016	33	527.42	531.87	92.59
nest 10	April 2016	24	912.96	1168.28	238.47
**Grand Total**		**159**	507.34	799.65	63.42

	date	Count of bee	time [log(s)] taken until first flower visitation Average	StdDev	SE
**control**		**65**	**1.75**	**0.80**	**0.10**
nest 3	March 2015	8	1.24	1.28	0.45
nest 4	March 2015	23	1.79	0.73	0.15
nest 8	March 2016	34	1.84	0.69	0.12
**imidacloprid**		**94**	**2.44**	**0.74**	**0.08**
nest 6	April 2015	37	2.39	0.85	0.14
nest 9	March 2016	33	2.49	0.52	0.09
nest 10	April 2016	24	2.45	0.82	0.17
**Grand Total**		**159**	**2.16**	**0.83**	**0.07**

	date	Count of bee	duration (s) of flowering period Average	StdDev	SE
**control**		**65**	**1394.46**	**1059.24**	**131.38**
nest 3	March 2015	8	1529.43	1391.30	491.90
nest 4	March 2015	23	1166.13	912.16	190.20
nest 8	March 2016	34	1524.97	1086.20	186.28
**imidacloprid**		**94**	**910.82**	**645.31**	**66.56**
nest 6	April 2015	37	700.53	437.25	71.88
nest 9	March 2016	33	1104.70	727.94	126.72
nest 10	April 2016	24	961.78	718.42	146.65
**Grand Total**		**159**	**1107.39**	**868.66**	**68.89**

	date	Count of bee	number of flowers visited Average	StdDev	SE
**control**		**65**	**14.94**	**5.44**	**0.67**
nest 3	March 2015	8	14.50	7.23	2.56
nest 4	March 2015	23	15.48	4.22	0.88
nest 8	March 2016	34	14.68	5.83	1.00
**imidacloprid**		**94**	**12.65**	**5.61**	**0.58**
nest 6	April 2015	37	13.16	5.07	0.83
nest 9	March 2016	33	13.18	4.16	0.72
nest 10	April 2016	24	11.13	7.74	1.58
**Grand Total**		**159**	**13.58**	**5.64**	**0.45**

	date	Count of bee	all flower types visited (0 = no, 1 = yes) Average	StdDev	SE
**control**		**65**	**0.55**	**0.50**	**0.06**
nest 3	March 2015	8	0.88	0.35	0.13
nest 4	March 2015	23	0.39	0.50	0.10
nest 8	March 2016	34	0.59	0.50	0.09
**imidacloprid**		**94**	**0.22**	**0.42**	**0.04**
nest 6	April 2015	37	0.22	0.42	0.07
nest 9	March 2016	33	0.21	0.42	0.07
nest 10	April 2016	24	0.25	0.44	0.09
**Grand Total**		**159**	**0.36**	**0.48**	**0.04**

	date	Count of bee	any blue (punishing) flowers visited (0 = no, 1 = yes)		
			Average	StdDev	SE
**control**		**65**	**0.58**	**0.50**	**0.06**
nest 3	March 2015	8	0.88	0.35	0.13
nest 4	March 2015	23	0.39	0.50	0.10
nest 8	March 2016	34	0.65	0.49	0.08
**imidacloprid**		**94**	**0.27**	**0.44**	**0.05**
nest 6	April 2015	37	0.24	0.43	0.07
nest 9	March 2016	33	0.21	0.42	0.07
nest 10	April 2016	24	0.38	0.49	0.10
**Grand Total**		**159**	**0.40**	**0.49**	**0.04**

	date	Count of bee	visitations	rewarding (1 = yellow) vs punishing (0 = blue & orange) flowers visited		
				Average	StdDev	SE (bee level)
**control**		**65**	**3003**	**0.84**	**0.36**	**0.05**
nest 3	March 2015	8	375	0.84	0.36	**0.13**
nest 4	March 2015	23	1177	0.90	0.30	**0.06**
nest 8	March 2016	34	1451	0.80	0.40	**0.07**
**imidacloprid**		**94**	**3641**	**0.87**	**0.33**	**0.03**
nest 6	April 2015	37	1731	0.93	0.26	**0.04**
nest 9	March 2016	33	1032	0.85	0.35	**0.06**
nest 10	April 2016	24	878	0.79	0.41	**0.08**
**Grand Total**		**159**	**6644**	**0.86**	**0.35**	**0.03**

Model diagnostics

1. Physical performance – all data with one value per bumblebee

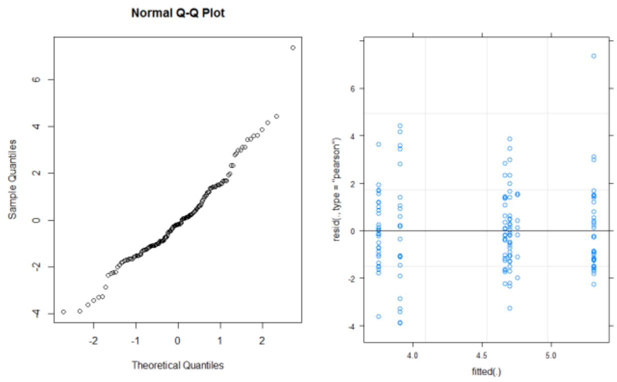

Fig. S3. QQ-plot and residuals of model 1.1 (average speed of movements between flowers (m/s))

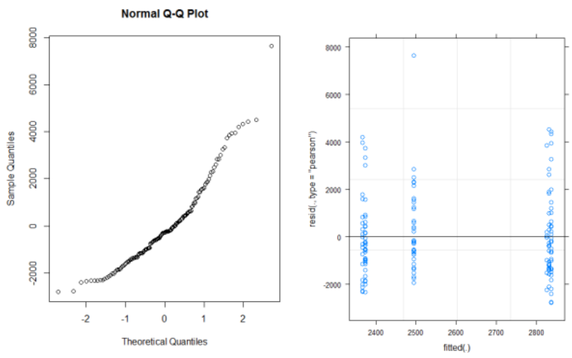

Fig. S4. QQ-plot and residuals of model 1.2 [total distance moved between flowers (cm)]

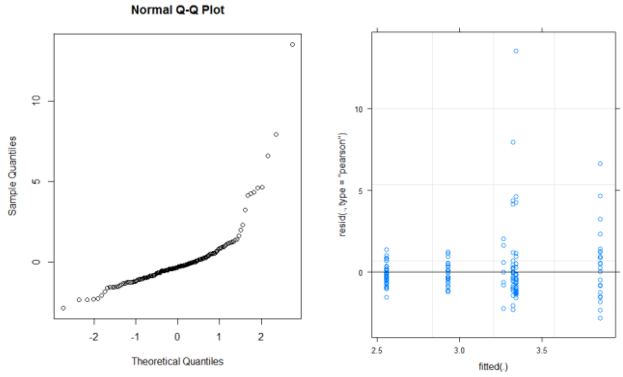

Fig. S5. QQ-plot and residuals of model 1.3 [average time (s) spent feeding].

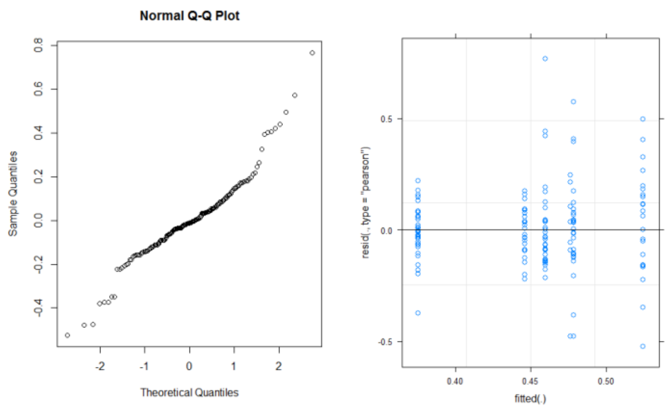

Fig. S6. QQ-plot and residuals of previous model 1.4 [average time log(s) spent feeding]. Basic diagnostic looks better and the results are the same (NS; *P*>0.05) with or without the log-transformation. Based on this data, we present the log-transformed version in Table 1.

2. Learning – all data with multiple values per bumblebee

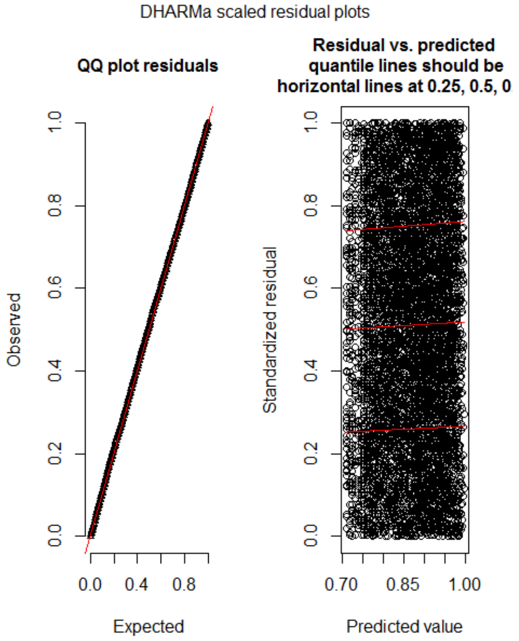

Fig. S7. Simulated residuals (using DHARMA (v-0.1.5)-package) of the model 2.1 (All visitations to rewarding (yellow) vs punishing (blue & orange) flowers**)**

The DHARMA-package produces Kolmogorov-Smirnov(K-S)-uniformity test for previous model **[**All visitation s to rewarding (yellow) vs punishing (blue & orange) flowers**].** The p-value is just under 0.05, which is not su rprising with such a large dataset (n=6644 visitations):

One-sample Kolmogorov-Smirnov test
D = 0.01724, p-value = 0.03853
alternative hypothesis: two-sided

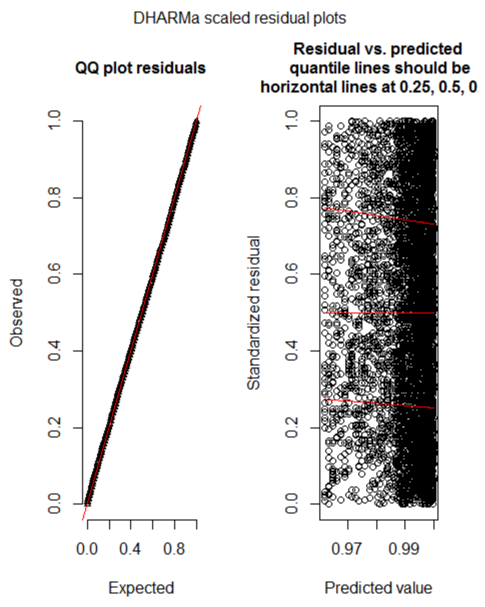

Fig. S8. Simulated residuals (using DHARMA-package) of the previous model 2.2 **(**easy discrimination task: yellow vs blue flowers**)**

The DHARMA-package produces K-S-uniformity test for previous model **(**easy discrimination task: yellow vs blue flowers**):**

One-sample Kolmogorov-Smirnov test:
D = 0.015011, p-value = 0.1408
alternative hypothesis: two-sided

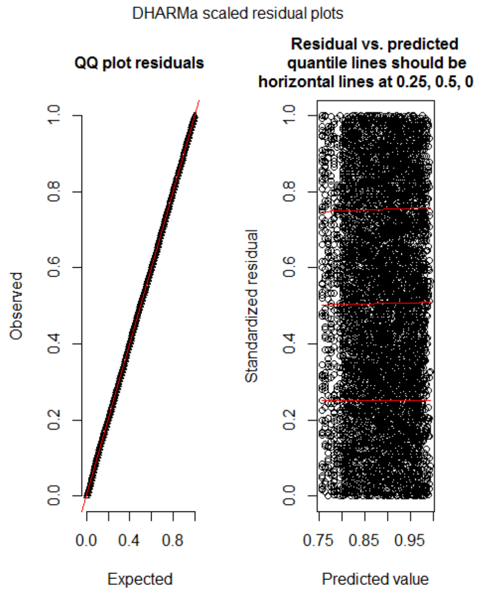

Fig. S9. Simulated residuals (using DHARMA-package) of the previous model 2.3 (difficult discrimination task: yellow vs orange flowers**)**

The DHARMA-package produces K-S-uniformity test for model 2.3 (difficult discrimination task: yellow vs or ange flowers):

One-sample Kolmogorov-Smirnov test:
D = 0.010062, p-value = 0.53
alternative hypothesis: two-sided

3. Foraging motivation – all data with one value per bumblebee

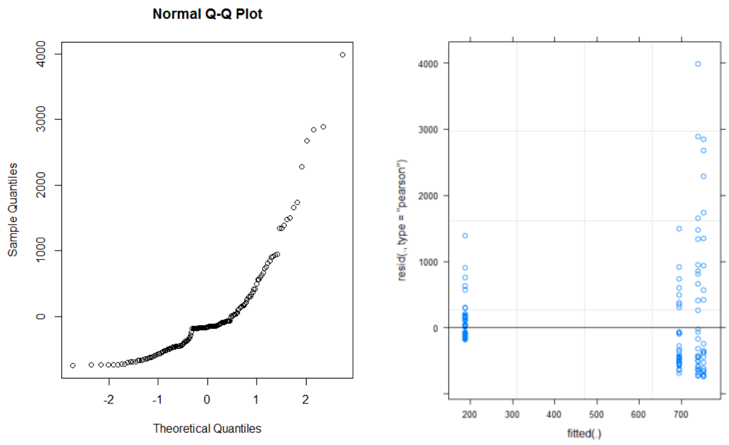

Fig. S10. QQ-plot and residuals of model 3.1 [time (s) taken until first flower visitation]. QQ-plot is skewed and residuals seem to increase with larger fitted values. Based on these diagnostics, we will fit a model with a log-transformation for the response variable.

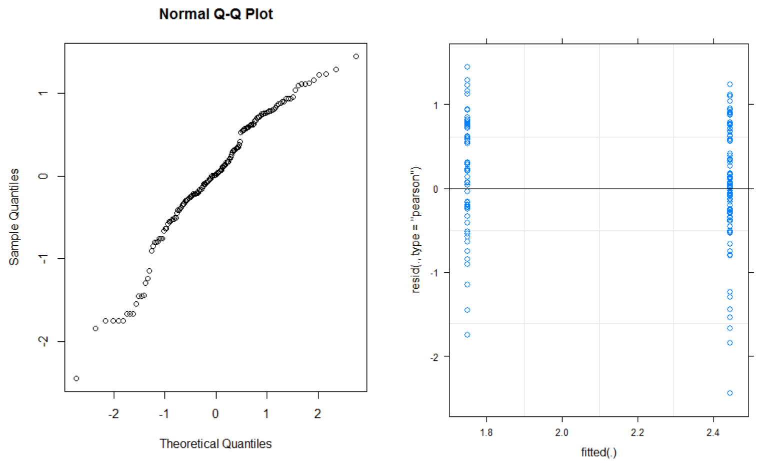

Fig. S11. QQ-plot and residuals of model 3.2 [time log(s) taken until first flower visitation]. QQ-plot looks less skewed and residuals are uniform with log-transformation. Results are similar ($p<0.05$) with or without the log-transformation, but a log-transformed model gives a better fit.

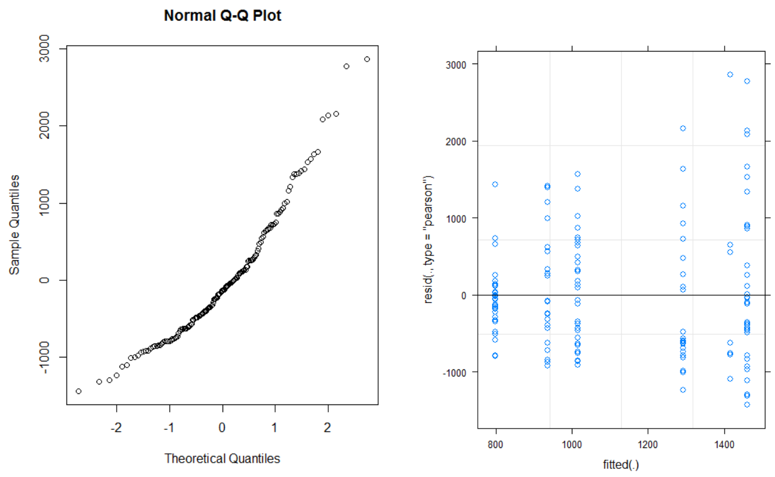

Fig. S12. QQ-plot and residuals of previous model 3.3 (duration (s) of foraging period). Residuals tend to increase marginally, but the model should be robust enough to be reliable. We did try log and square root-transformations, but as the results (p-values for treatment; 0.084 & 0.068 respectively) and diagnostic figures were similar, so we kept the non-transformed model in the main results.

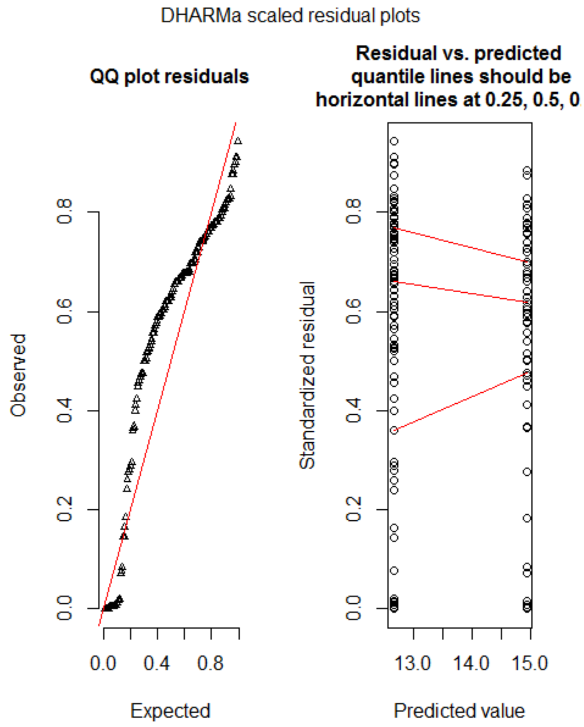

Fig. S13. Simulated residuals (using DHARMA-package) of model 3.5 (number of flowers visited – negative binomial distribution**).** The QQ-plot is not so meaningful for counts-data, but the simulated residuals seem to be non-uniformal.

The DHARMA-package produces K-S-uniformity test for previous model (number of flowers visited):
One-sample Kolmogorov-Smirnov test:

D = 0.21069, p-value = 1.48e-06

alternative hypothesis: two-sided

The model (number of flowers visited) with negative binomial distribution seems to be suffering from poor fit. There is no overdispersion issues, however:

DHARMa nonparametric overdispersion test via IQR of scaled residuals against IQR expected under uniform

dispersion = 0.60217, p-value = 1
alternative hypothesis: overdispersion

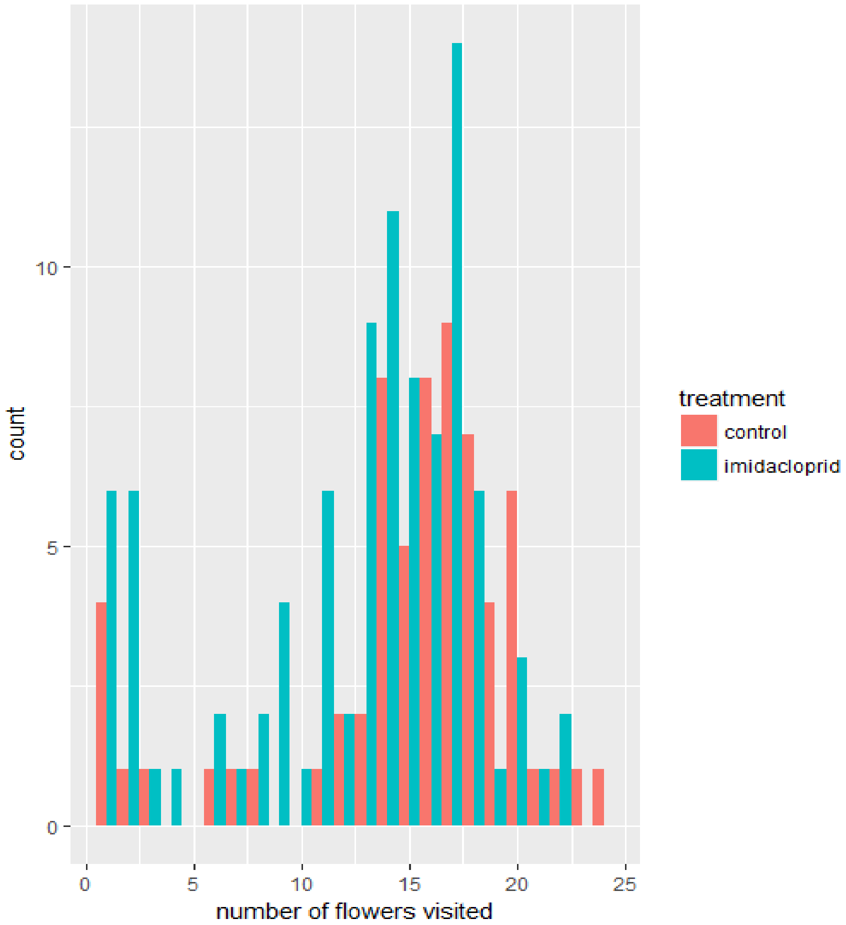

Fig. S15. Problems with the poor fit seem to be related to data distribution. The data seems to be bimodal, meaning that in addition to the main peak, there is another peak with less than five flowers visited.

We tested the probability of having over five flowers visited (1 = more than five; 0 = less than five flowers visited) by treatment:

Model 3.6: *over five flowers visited* (1 = more than five; 0 = less than five flowers visited)

Generalized linear mixed model fit by maximum likelihood (Laplace Approximation) [
glmerMod]
 Family: binomial (logit)
Formula: over5cat ~ treatment + (1 | nest)
   Data: dataset2

AIC BIC logLik deviance df.resid

123.9	133.1	-59.0	117.9	156

Scaled residuals:

Min	1Q	Median	3Q	Max
-3.5584	0.2810	0.3430	0.3794	0.5526

Random effects:

Groups	Name	Variance	Std.Dev.
nest	(Intercept)	0.2473	0.4973

Number of obs: 159, groups: nest, 6

Fixed effects:

	Estimate	Std. Error	z value	Pr(>|z|)
(Intercept)	2.3696	0.5414	4.377	1.2e-05 ***
treatmentimidacloprid	-0.5939	0.6764	-0.878	0.38

Signif. codes: 0 '***' 0.001 '**' 0.01 '*' 0.05 '.' 0.1 ' ' 1

Correlation of Fixed Effects:

	(Intr)
trtmntmdclp	-0.788

There is no statistically significant difference between treatments based on the p-value of previous model in the likelihood of having more than five flowers visited. Next we tried if model assumptions are met if we excluded the bimodality by removing count values less than five. Results are similar:

Model 3.7: *number of flowers visited* – values less than five removed

Generalized linear mixed model fit by maximum likelihood (Laplace Approximation) [
glmerMod]
Family: Negative Binomial(748703.3) (log)
Formula: over5counts ~ treatment + (1 | nest)
Data: dataset2

AIC	BIC	logLik	deviance	df.resid
757.6	769.3	-374.8	749.6	135

Scaled residuals:

Min	1Q	Median	3Q	Max
-2.5520	-0.4063	0.1180	0.6423	1.9531

Random effects:

Groups	Name	Variance	Std.Dev.
nest	(Intercept)	2.662e-14	1.632e-07

Number of obs: 139, groups: nest, 6

Fixed effects:

	Estimate	Std. Error	z value	Pr(>|z|)
(Intercept)	2.79148	0.03198	87.30	< 2e-16 ***
treatmentimidacloprid	-0.11389	0.04317	-2.64	0.00833 **

Signif. codes: 0 ‘***’ 0.001 ‘**’ 0.01 ‘*’ 0.05 ‘.’ 0.1 ‘ ’ 1

Correlation of Fixed Effects:
(Intr)
trtmntmdclp -0.736

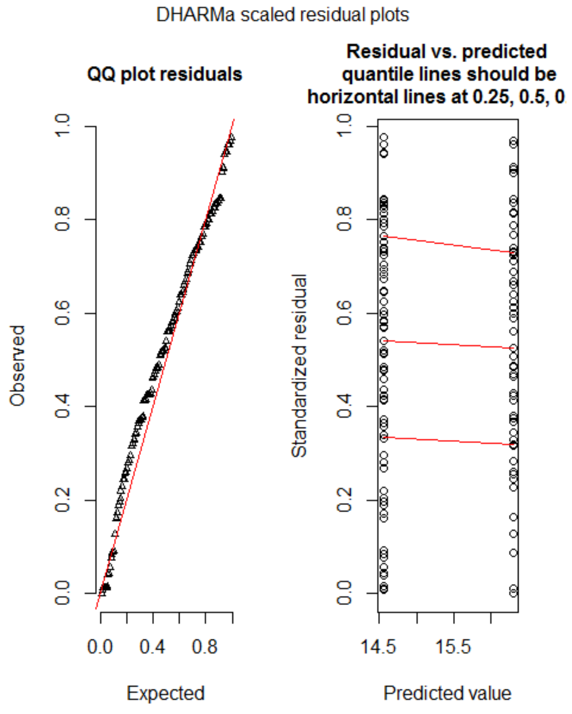

Fig. S16. Diagnostics from the model 3.7 above (number of flowers visited, values less than 5 removed). Residuals indicate a better fit.

The DHARMA-package produces K-S-uniformity test for previous model (number of flowers visited, values l ess than five are excluded from data):

DHARMa nonparametric overdispersion test via IQR of scaled residuals against IQR expected under unifor m:

dispersion = 0.8407, p-value = 0.967
alternative hypothesis: overdispersion

If the bimodality (“lazy” bumblebees) are removed, K-S-test finally gives p-values greater than 0.05. Model f its with the assumed distribution:

One-sample Kolmogorov-Smirnov test:
D = 0.088259, p-value = 0.229
alternative hypothesis: two-sided

Conclusion of diagnostics on number of flowers visited:

Which option is better, removing data from results or not meeting all model assumptions? We decided it is better to have data from all bumblebees presented in main results and not remove those “lazy” bumblebees. The GLMM models are quite robust and the results are the same (bumblebees visited fewer flowers in imidacloprid treatment, $p<0.05$) with or without the bimodality.

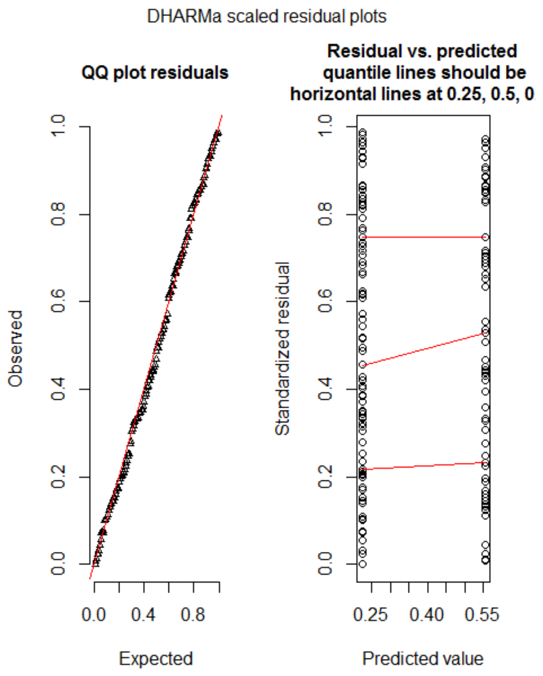

Fig. S17. Simulated residuals (using DHARMA-package) of the model 3.8 (all flower types visited (0 = no, 1 = yes)**)**

The DHARMA-package produces K-S-uniformity test for previous model **(**all flower types visited (0 = no, 1 = yes), model seems to fit with the assumed distribution:

One-sample Kolmogorov-Smirnov test:
D = 0.037937, p-value = 0.9761
alternative hypothesis: two-sided

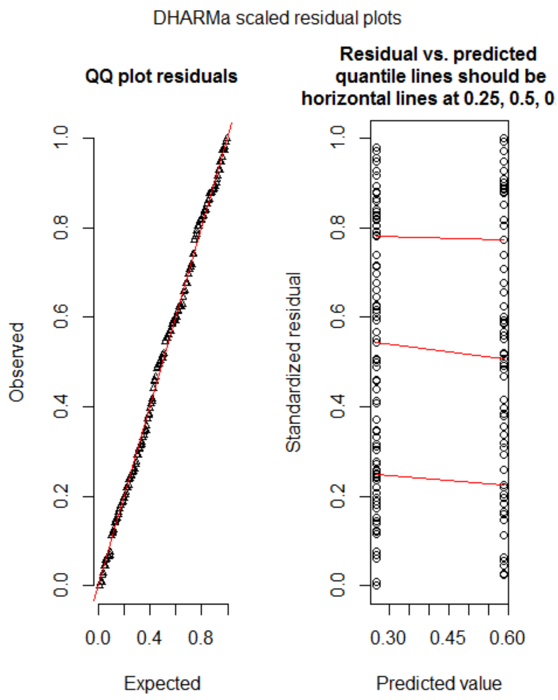

Fig. S18. Simulated residuals (using DHARMA-package) of the model 3.9 [any blue (punishing) flowers visited (0 = no, 1 = yes)]

The DHARMA-package produces K-S-uniformity test for previous model **(**any blue (punishing) flowers visited (0 = no, 1 = yes)):

One-sample Kolmogorov-Smirnov test:

D = 0.037459, p-value = 0.9789
alternative hypothesis: two-sided

There seems to be no overdispersion in the previous model (any blue (punishing) flowers visited (0 = no, 1 = yes):

Chisq test for overdispersion in GLMMs

dispersion = 1.0027, pearSS = 156.4300, rdf = 156.0000, p-value = 0.4753
alternative hypothesis: true dispersion greater 1
